# Preclinical Models of Craniospinal Irradiation for Medulloblastoma

**DOI:** 10.3390/cancers12010133

**Published:** 2020-01-05

**Authors:** Jennifer L. Stripay, Thomas E. Merchant, Martine F. Roussel, Christopher L. Tinkle

**Affiliations:** 1 Departments of Tumor Cell Biology, St. Jude Children’s Research Hospital, Memphis, TN 38105, USA; jennifer.stripay@stjude.org (J.L.S.); martine.roussel@stjude.org (M.F.R.); 2 Departments of Radiation Oncology, St. Jude Children’s Research Hospital, Memphis, TN 38105, USA; thomas.merchant@stjude.org

**Keywords:** craniospinal irradiation, radiation therapy, medulloblastoma, animal models, preclinical, translational, patient-derived orthotopic xenografts

## Abstract

Medulloblastoma is an embryonal tumor that shows a predilection for distant metastatic spread and leptomeningeal seeding. For most patients, optimal management of medulloblastoma includes maximum safe resection followed by adjuvant craniospinal irradiation (CSI) and chemotherapy. Although CSI is crucial in treating medulloblastoma, the realization that medulloblastoma is a heterogeneous disease comprising four distinct molecular subgroups (wingless [WNT], sonic hedgehog [SHH], Group 3 [G3], and Group 4 [G4]) with distinct clinical characteristics and prognoses has refocused efforts to better define the optimal role of CSI within and across disease subgroups. The ability to deliver clinically relevant CSI to preclinical models of medulloblastoma offers the potential to study radiation dose and volume effects on tumor control and toxicity in these subgroups and to identify subgroup-specific combination adjuvant therapies. Recent efforts have employed commercial image-guided small animal irradiation systems as well as custom approaches to deliver accurate and reproducible fractionated CSI in various preclinical models of medulloblastoma. Here, we provide an overview of the current clinical indications for, and technical aspects of, irradiation of pediatric medulloblastoma. We then review the current literature on preclinical modeling of and treatment interventions for medulloblastoma and conclude with a summary of challenges in the field of preclinical modeling of CSI for the treatment of leptomeningeal seeding tumors.

## 1. Introduction

Medulloblastoma is a posterior fossa embryonal tumor of neuroectodermal origin that is thought to arise from progenitor cells present during early brain development [1,2,3]. It is the most common malignant brain tumor in children and adolescents in the United States [4]. For most patients, optimal management of medulloblastoma includes maximum safe resection followed by adjuvant craniospinal irradiation (CSI) and chemotherapy. For very young children, defined as those younger than 3 years at diagnosis, chemotherapy with or without focal tumor-bed irradiation is often used to delay or avoid CSI [5]. Advances in molecular pathology have revealed significant heterogeneity within this cerebellar tumor and have identified four distinct subgroups (wingless [WNT], sonic hedgehog [SHH], Group 3 [G3], and Group 4 [G4]). This has significant implications for future applications of surgery, chemotherapy, and radiation therapy (RT) [6]. In this review, we first outline the current clinical indications for irradiation of pediatric medulloblastoma and the technical aspects of this treatment approach. We then review the current literature on preclinical modeling of, and treatment interventions for, medulloblastoma. The review concludes with a summary of outstanding questions in the field of preclinical modeling of RT for pediatric brain tumors and a consideration of future directions for treating leptomeningeal seeding tumors with these approaches.

## 2. Clinical Management of Medulloblastoma: The Evolving Role of Radiation Therapy

### 2.1. Background

Like many embryonal tumors, medulloblastoma shows a predilection for distant metastatic spread and leptomeningeal seeding, with subarachnoid dissemination present in approximately 20% to 35% of cases at diagnosis and in most cases at relapse [7,8]. CSI targeting the entire neuraxis has been a standard-of-care therapy for most patients. As cure became a reality, however, long-term survivors were noted to suffer from significant radiation-associated late effects, including neurocognitive and neuroendocrine deficits, bone and soft tissue hypoplasia, vascular insults, and subsequent malignant neoplasms. Thus, efforts have been directed toward maintaining or improving overall survival rates while minimizing treatment-related toxicity. These efforts have primarily focused on modifications to RT approaches that can be broadly grouped as follows: investigations of the effects of adding concurrent and/or adjuvant chemotherapy regimens [9,10]; investigations of the effects of delaying or omitting RT for very young children [11,12,13,14]; investigations of the effects of reductions in the RT dose and field size [15,16]; and, over the past decade, the identification of distinct molecular subgroups of medulloblastoma with specific alterations that drive tumorigenesis, facilitating the identification of new therapeutic targets and enhanced risk stratification [17,18,19].

### 2.2. Defining the Role of Radiotherapy across Clinical Risk Groups of Medulloblastoma

The evolution of RT for medulloblastoma has principally involved delaying or omitting RT in very young patients and reducing RT dose and target volumes in older patients. In the past, patients with medulloblastoma, irrespective of age or tumor characteristics, were treated to high doses to the craniospinal axis, with doses ranging from 36 to 40 Gy, followed by focal boost to the posterior fossa for a cumulative dose of 54 to 60 Gy [20,21]. Devastating neurocognitive sequelae after RT, particularly in very young children [22,23,24], spurred the use of surgery and chemotherapy alone or with delayed CSI approaches in very young patients (i.e., those younger than 3 or 5 years, depending on the study), and these approaches have subsequently evolved into the standard of care treatment [25,26,27,28,29,30]. The role of primary tumor site-only irradiation in very young patients continues to be evaluated, and reports have been mixed as to the potential impact of this approach with respect to improving survival outcomes [31,32,33]. Subsequent attempts have focused on CSI dose reductions in patients older than 3 to 5 years with “standard-risk” medulloblastoma, generally defined as nonmetastatic disease without significant residual primary tumor after surgery [21,34,35]. However, an initial attempt through the Pediatric Oncology Group (POG), in which CSI doses were lowered from 36 to 23.4 Gy for such standard-risk patients, resulted in an increased risk of early relapse, early isolated neuraxis relapse, and lower 5-year event-free survival and overall survival [36]. The Children’s Cancer Group (CCG) conducted a series of studies exploring the role of chemotherapy in combination with CSI as an alternative approach to CSI dose de-escalation. These studies demonstrated that in standard-risk patients treated with reduced-dose CSI to 23.4 Gy and posterior fossa boost to 55.8 Gy concurrent with vincristine followed by adjuvant lomustine, vincristine, and cisplatin, survival rates were comparable to those observed in historical studies of standard-risk patients treated with high-dose CSI and boost RT alone [9,10,21]. Importantly, neurocognitive function appeared to be better preserved with reduced-dose CSI and chemotherapy relative to that seen in patients treated with high-dose CSI alone [37].

Notably, these studies did not directly compare reduced-dose CSI in combination with chemotherapy to high-dose CSI and boost RT alone in standard-risk patients specifically. The only randomized trial in which this was done, in fact, showed that adding chemotherapy to RT had a negative impact on survival in standard-risk patients [38]. That said, subset analyses of data from these randomized trials have consistently suggested that chemotherapy in combination with RT improves survival in high-risk patients [21,39]. Given the ability to administer lower doses of radiation to standard-risk patients and the results observed in high-risk patients, chemotherapy has been widely adopted in the management of patients with medulloblastoma at standard or high risk of relapse. Of note, approaches in which chemotherapy was delivered after surgery and before RT, and in which supratentorial radiation was omitted, resulted in inferior outcomes [38,40].

Reductions in the primary-site boost volume have also been evaluated. Initial retrospective pattern-of-failure studies suggested a low incidence of isolated posterior fossa recurrence outside the primary tumor bed. Subsequent pilot studies demonstrated the feasibility of reducing boost fields from the entire posterior fossa to the more limited postoperative surgical bed [41,42,43]. Merchant and collaborators prospectively studied the use of a 2 cm clinical target volume (CTV) margin targeting the post-operative tumor bed during boost treatment (after an initial 36 Gy to the entire posterior fossa) and demonstrated a cumulative posterior fossa failure rate of 4.9% in standard-risk patients [16]. Building on these findings, the Children’s Oncology Group (COG) launched the randomized phase 3 clinical trial ACNS0331 in 2004, in which standard-risk patients aged 3 to 7 years were randomized to 23.4 vs. 18 Gy of CSI and all patients were randomized to boost RT to the entire posterior fossa vs. a primary tumor bed boost with a 1.5 cm CTV margin. Preliminary results, presentation at the American Society of Radiation Oncology (ASTRO) meeting in 2016, showed no significant differences in event-free survival or overall survival with different boost volumes [44]. Disappointingly, reduced-dose CSI to 18 Gy was associated with an increased distant failure rate and significantly inferior survival outcomes, with a 5 year distant failure rate of 12.8% vs. 8.2%, event-free survival of 72.1% vs. 82.6%, and overall survival of 78.1% vs. 85.9%, for 18 and 23.4 Gy of CSI, respectively. Thus, the standard-risk CSI dose in the United States continues to be 23.4 Gy. For patients with high-risk disease, the CSI dose remains 36 Gy with a posterior fossa boost to 54–55.8 Gy. In an effort to improve outcomes for high-risk patients, the ACNS0332 randomized trial [45] addressed the potential radiosensitizing effect of adding carboplatin to vincristine concurrently with CSI to 36 Gy followed by posterior fossa boost to 55.8 Gy and maintenance chemotherapy; the study recently closed, and results are pending. Ultimately, defining outcomes across the distinct molecular subgroups of medulloblastoma will be critically important to fully evaluate the utility of these and future reduced dose–volume and radiosensitizing approaches.

### 2.3. Towards Subgroup-Specific Radiotherapy Guidelines

Based primarily on DNA methylation analysis, medulloblastoma is known to be a heterogeneous disease comprising four distinct molecular subgroups (WNT, SHH, G3, and G4) with significant differences in their clinical and pathologic characteristics, genetic drivers of disease development and progression patterns, and clinical prognosis [18,46,47,48]. More recent studies employing additional genomic analyses and patient samples have identified distinct subtypes within each of these molecular subgroups [19,49,50,51]. These data have already informed clinical trial design, with the incorporation of molecular profiling to help guide dose de-escalation in WNT medulloblastoma and intensify therapy in G3 medulloblastoma. For example, following preclinical work demonstrating improved survival in models of high-risk G3 medulloblastoma with the addition of gemcitabine and pemetrexed [52], a St. Jude Children’s Research Hospital trial, SJMB12 [53], is evaluating whether adding this regimen to the standard therapy for patients with high-risk G3/G4 medulloblastoma will improve outcomes. Additionally, the reduction of the CSI dose from 23.4 to 15 Gy and the tumor bed boost dose from 55.8 to 51 Gy is being evaluated in patients on this study with standard-risk WNT medulloblastoma, based on the consistently observed excellent outcomes of WNT medulloblastoma and a review of the patterns-of-failure dosimetry following conventional CSI doses in these patients. Cohen and colleagues evaluated a more aggressive approach to therapy de-escalation in these patients, in which RT was omitted altogether for patients with nonmetastatic, completely resected WNT medulloblastoma [54]. Unfortunately, this study was closed early because of progressive disease in the cranium and spine in the first two patients enrolled. The concomitant failure in the spine highlights the necessity of continued spinal irradiation in the setting of current cytotoxic chemotherapy. However, the optimal prophylactic CSI dose remains to be determined. To this end, the recently activated COG ACNS1422 trial is evaluating a CSI dose of 18 Gy followed by a primary-site boost to 54 Gy as well as a de-escalated chemotherapy approach with omission of vincristine during CSI and reduced-dose maintenance chemotherapy for standard-risk WNT medulloblastoma [55]. The impact of the molecular subgroups within medulloblastoma may also apply to outcomes after surgical resection, with a recent study demonstrating more aggressive surgery beyond subtotal resection does not affect progression-free survival for patients with WNT, SHH, or G3 tumors [56]. Finally, recent data suggest that neurocognitive outcomes may also vary by medulloblastoma subgroup, hinting that treatment de-escalation may not have a uniform impact on the risk of late effects for all patients with medulloblastoma [57].

### 2.4. Advances in Radiotherapy Techniques

**Figure 1:**
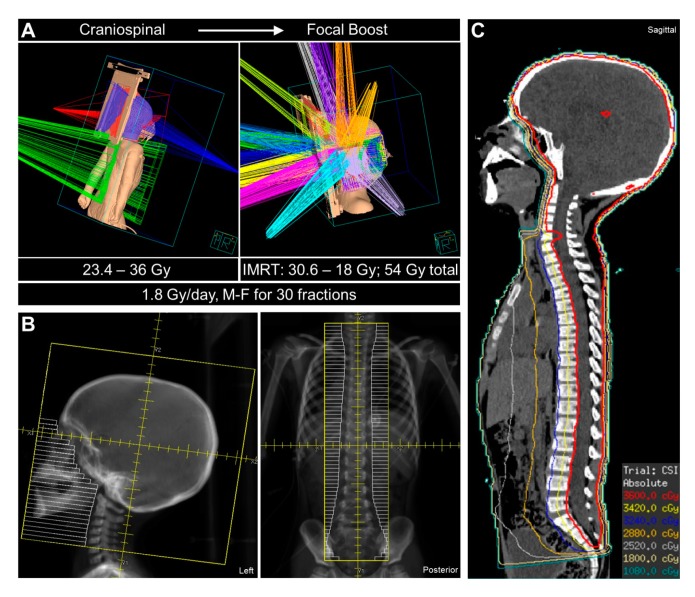
Clinical radiation therapy for medulloblastoma. (**A**) Three-dimensional rendering of craniospinal irradiation (CSI) (left) in a pediatric patient with medulloblastoma treated in the supine position with parallel-opposed lateral beams (red, purple) and a single posterior-to-anterior (PA) beam (green) and with intensity-modulated radiation therapy (IMRT)-based primary tumor site boost irradiation with multiple beams (right). Typical CSI and boost doses and fractionation patterns are noted. M–F, Monday through Friday. (**B**) Beam’s eye view of the cranium (left) and spine (right) CSI fields on digitally reconstructed radiographs (DRRs), with multileaf collimators depicted in white. (**C**) CSI isodose distributions overlaid on the sagittal planning computed tomography (CT) scan.

Irradiation techniques and delivery modalities for CSI have also evolved. Multiple studies have demonstrated the critical importance of ensuring adequate coverage of the entire target volume, encompassing the meninges covering the brain and spinal cord, including extensions along the nerve roots, with more frequent relapses and inferior survival being observed in patients treated with CSI with targeting deviations [58,59,60]. Thus, current standard techniques employ computed tomography (CT)-based target delineation, often with the supplemental use of magnetic resonance imaging (MRI) through image registration techniques, and image-guided RT, usually in the form of planar X-rays or cone-beam CT, to ensure interfraction reproducibility. CSI has historically employed megavoltage photons delivered through a simple geometric beam arrangement of parallel-opposed lateral fields to treat the cranium and a single posterior-to-anterior (PA) beam to treat the spine, with collimation of the beams to the contours of the brain and spine (Figure 1). For the boost phase of treatment, conformal approaches are now generally used, most commonly employing intensity-modulated radiotherapy (IMRT) in which multiple beams are used, each with its dose distribution modulated through multileaf collimation. Long-term studies have demonstrated significant reductions in grade 3/4 ototoxicity when an IMRT boost is used, as compared to the historical treatment approach with two lateral fields [61,62]. Variations on IMRT, in which irradiation is delivered as the gantry moves, including volumetric modulated arc therapy (VMAT) and helical tomotherapy, have also been used to enhance the conformality of CSI, as well as the primary-site boost irradiation [63,64]. More recently, proton therapy has emerged as a major RT modality for the treatment of medulloblastoma. Multiple dosimetric studies of RT for medulloblastoma have demonstrated the superior dose distribution of protons as compared to photons [65], and emerging clinical evidence suggests that proton-based CSI/boost therapy for pediatric patients with medulloblastoma reduces acute and late effects without reducing tumor control [66,67,68,69,70].

## 3. Preclinical Modeling of CSI in Medulloblastoma

### 3.1. Current Models of Medulloblastoma

Developing new and more effective therapeutic approaches to treating pediatric medulloblastoma requires the use of accurate preclinical models to inform clinical trial design. Several groups have developed murine models of medulloblastoma, primarily in an effort to create preclinical testing tools. These models range from genetically engineered murine models (GEMMs) to patient-derived orthotopic xenografts (PDOXs) [71]. Next-generation sequencing technologies have provided insights into the molecular underpinnings of medulloblastoma subgroups, and all four subgroups have been modeled in murine systems and with patient-derived material [71,72,73]. Most preclinical testing has focused on high-risk SHH and G3 tumors, for which the need for improved therapies is greatest [18].

### 3.2. Preclinical Pipeline Limitations and Considerations

Medulloblastoma presents unique challenges with regard to preclinical modeling and therapeutic evaluation. The genetic and molecular heterogeneity of the disease dictates the use of multiple in vitro and in vivo models to evaluate response variability. Interrogation of novel and optimized therapies has been conducted in cell lines or xenograft models derived from naïve patient biopsies or in mouse models of primary disease. These experimental therapies are, thus, delivered “at diagnosis” and not in a recurrent setting after standard therapy. Although upfront treatment approaches are certainly under evaluation, most clinical trials enroll patients who have already undergone surgery, chemotherapy, and/or RT. Another assumption often made in preclinical testing is that the biology of recurrent disease is largely similar to that of the tumor at primary diagnosis. Work by Taylor and colleagues revealed that medulloblastoma recurrence is driven largely by clonal selection and that dominant clones present at recurrence may contain molecular targets that differ considerably from those in clones that predominate at diagnosis [74]. Medulloblastoma subgroups also vary dramatically in their timing and pattern of recurrence. To better inform molecularly stratified trials, a more complete understanding of therapeutic response trends in these subgroups will be critical. Most experimental preclinical models have failed to model recurrent disease, which may account for the lack of efficacy of treatment in many clinical trials.

Analysis of in vitro and in vivo preclinical studies of radiation-related interventions suggests that improving their efficacy will require the incorporation of standard-of-care modalities that use clinically relevant and carefully delivered RT regimens [75]. Optimizing radiation delivery in a preclinical setting has been challenging, but several investigators have developed approaches to evaluate the therapeutic response by using available technologies, including the small animal radiation research platform (SARRP^®^) micro-irradiator (XStrahl, Inc.) and the X-Rad 225Cx image-guided small animal irradiator (Precision X-Ray, Inc.) [76,77]. Both systems use volumetric cone-beam CT images to visualize bone or soft tissues, to place treatment isocenters, and to enable RT dose calculation and assessment of the 3D dose distribution. This image-guided approach facilitates treatment of the entire neuroaxis, with treatment-field isocenters being positioned in both the brain and the spinal cord. The primary endpoint of prior studies using these technologies has been overall survival in tumor-bearing animals, but each of those investigations had some unique features in terms of the study design, radiation administration, and response evaluation.

### 3.3. Existing Models of Craniospinal Irradiation

The first study to evaluate image-guided delivery of radiation in a preclinical setting for medulloblastoma was conducted by Taylor and colleagues [74]. Using the X-Rad 225CX system equipped with a fixed collimator, they employed CSI after surgical resection of the primary tumor to mimic upfront therapy in patients and model recurrent disease. They used a GEMM of SHH medulloblastoma and enrolled animals on the study once clinical signs of neurologic impairment were observed. Mice were subjected to partial tumor resection and allowed to recover before undergoing irradiation. CSI was administered in fractionated doses over the course of 4 weeks (with spinal irradiation being introduced in week 2) to achieve an exposure of 36 Gy, which is comparable to the clinical dose used for high-risk patients. The blinded study revealed significant improvement in overall survival in mice that underwent resection and irradiation, with 40% of the mice being free of disease on long-term follow-up. The remainder of the mice in the study cohort developed either local or metastatic relapse, and subsequent analysis found that genetic events common to both therapy-naïve and recurrent tumors were rare. Ultimately, this work revealed that there was clonal selection of molecular and genetic events in tumors after surgery and irradiation and recurrent convergence on individual signaling pathways. These findings raise the possibility of anticipatory therapy, whereby drivers of therapeutic resistance would be targeted during the initial treatment.

**Figure 2:**
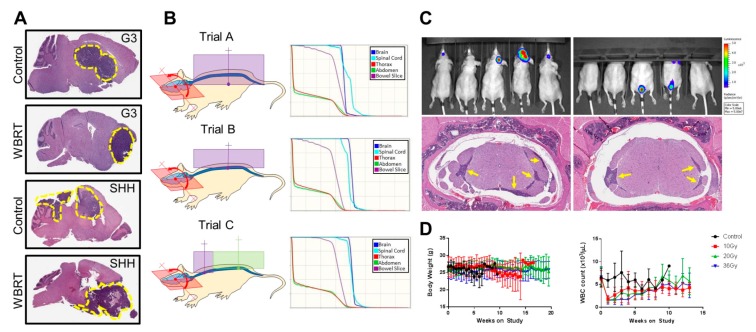
Preclinical modeling of craniospinal irradiation (CSI) of patient-derived orthotopic xenograft (PDOX) models of medulloblastoma. (**A**) Evidence of olfactory bulb metastasis in orthotopic xenograft mouse models of very-high-risk medulloblastoma when treated with non-image-guided fractionated cranial radiation therapy. Metastasis is indicated by dashed lines on sections stained with hematoxylin and eosin. (**B**) Schematic of three CSI treatment plans using the small animal radiation research platform (SARRP^®^) (left) and the associated dose (Gy; x-axis)–volume (percent; y-axis) histograms of indicated regions of interest (right). All mice received brain arc treatment (−90°, 90°), but the spinal fields were varied by number of fields and isocenter placement to determine the optimal treatment plan. (**C**) Upper panels: Pattern-of-failure analysis using custom head shielding to increase spinal bioluminescence sensitivity, with spinal metastasis indicated in two mice in the far-right panel. Lower panels: Arrows indicate spinal metastasis on sections stained with hematoxylin and eosin. (**D**) Body weight and hematologic toxicity of murine PDOX CSI demonstrating tolerability with expected transient depressions in white blood cells. (Reprinted with permission from “Preclinical Modeling of Image-Guided Craniospinal Irradiation for Very-High-Risk Medulloblastoma”, Smith, et al. 2011 [78]; Elsevier publisher. All rights reserved.).

Subsequent work from our group focused on the preclinical incorporation of CSI in the context of PDOX models of medulloblastoma, with the ultimate goal of evaluating adjuvant therapies [78]. PDOX models of very-high-risk SHH medulloblastoma (*MYCN* amplified, *TP53* mutation) and G3 medulloblastoma (*MYC* amplified) [18] labeled with firefly luciferase were generated in mice, which were monitored for tumor burden by bioluminescence imaging (BLI). The study enrollment threshold was set at 10^6^ photons/s, which corresponds with histologically identifiable tumor burden but no overt clinical symptoms or detection by MRI in vivo. The intent was to model minimal disease burden, as in a patient who had undergone tumor resection. CSI proved superior to whole-brain irradiation with regard to tumor coverage and recurrence patterns, highlighting the necessity of encompassing the entire neuraxis and the importance of image guidance in facilitating this (Figure 2). Iterative fractionated CSI treatment plans and delivery in non-tumor-bearing mice revealed CSI delivery to be optimal when using a single arc-based cranial field and two spinal fields, together with a PA beam arrangement with three isocenters (one brain and two spinal), to deliver radiation over 3.5 weeks in 2 Gy fractions to achieve a cumulative CSI dose of 36 Gy. A −90° to +90° arc was used for the cranial field to maximize sparing of the upper aerodigestive tract, whereas the two spinal fields were needed to account for the significant extension over the cervical spine in the mouse, which presented challenges regarding isocenter placement and the resulting dose distributions. Within this paradigm, we evaluated toxicity by monitoring body weight and blood counts and chemistries. We found that, except for the expected transient acute leukopenia and neutropenia, body weight and other lab values were not significantly affected by the treatment and no clinically detectable lasting effects on other systemic organs were identified. To determine the optimal CSI dose with which to evaluate combination therapy with systemic agents, mice were subjected to 10, 20, or 36 Gy of CSI delivered in 2 Gy daily fractions from Monday to Friday and were monitored for tumor progression (by BLI) and overall survival. The intent was to establish a non-curative CSI dose that would significantly increase overall survival, as observed clinically, while facilitating the evaluation of the potentiation of radiation by one or more systemic agents. In every medulloblastoma model studied, 36 and 20 Gy of CSI resulted in a significant survival advantage and minimal tumor recurrence, thus establishing an effective “curative” dosing regimen. In contrast, a cumulative CSI dose of 10 Gy delivered over 1 week extended the lifespan of mice harboring an SHH PDOX (by approximately 40 days) and of those harboring a G3 PDOX (by approximately 90 days), but all of these mice subsequently experienced tumor recurrence and ultimately succumbed to progressive disease.

More recent work by Huang et al. incorporated CSI and boost irradiation in a preclinical setting to explore potential combination therapy in G3 and G4 medulloblastoma PDOX models [79]. Using the RS-2000 Biological Irradiator (Rad Source Technologies) and custom-made lead shielding, they administered CSI in 2 Gy fractions over 5 days, followed by an additional 10 Gy delivered over 5 days specifically to the tumor injection site within the cerebellum. For boost-site delivery, the diameter of the lead aperture was made approximately 2 mm larger than the anticipated tumor size based on prior whole-brain histologic analysis after tumor implantation. The authors observed a significant survival advantage of 65 days with radiation alone in mice with a G3 PDOX, as compared to untreated controls. Interestingly, after identifying cardiac glycosides as potent inhibitors of medulloblastoma growth by using an in silico systems biology approach with in vitro confirmation, the authors found that treatment with single-agent digoxin resulted in an extension of survival similar to that observed with 20 Gy of irradiation; however, clear potentiation of this repurposed small molecule with RT was not observed. Table 1 summarizes select therapy and medulloblastoma model characteristics of these studies.

## 4. Outstanding Questions and Future Directions

Incorporating RT into the preclinical pipeline of medulloblastoma models will be critical to translational efforts to define novel therapeutic approaches for this disease, particularly for the highest-risk subsets, for which overall survival rates remain disappointingly low. Although limited in their scope, the studies performed to date have provided valuable insights into study design for these therapies and have raised important questions (Table 2).

The strong response to CSI in murine PDOX models is consistent with the positive responses in patients after upfront therapy. However, in several preclinical medulloblastoma models, clinically comparable CSI doses appear to be curative, with no tumor burden or disease progression being detected for hundreds of days after the completion of treatment. One important consideration is that the low-energy photons characteristic of small animal irradiators may exert a more substantial biological effect than the particles from clinic-based high-energy sources. In addition, there may be significantly fewer tumor clonogens, or tumor stem cells, within any orthotopic xenograft than in human patients; if this is the case, a “murine-equivalent” CSI dosing regimen will be much different from that currently employed clinically. This also raises critical questions for translational brain tumor research in general: What is the optimal tumor burden at the start of preclinical survival or tumor growth-delay studies, and what is the best modality for determining enrollment threshold (BLI, MRI, histopathology, etc.) (discussed below) [75,80,81]? Additionally, evidence of the importance of the tumor microenvironment in governing the response to radiation continues to emerge [82]), highlighting an obvious shortcoming of PDOX models.

Outstanding questions also remain regarding boost treatment. Preclinical studies employing CSI in medulloblastoma models have not routinely incorporated a boost phase of radiation after the CSI. However, patients aged 3 years or older most commonly receive wide-field radiation encompassing the brain and spine followed by a local boost to the tumor bed, whereas tumor-bed irradiation alone has been evaluated in younger children. Therefore, it is important to evaluate preclinically the role played by the additional boost RT or focal boost therapy alone. Our group has initiated studies to determine the value of an acute boost of irradiation after chronic treatment, but the spatial and temporal control of the delivery is not yet optimized, in part because of the enhanced RT response observed in our PDOX models. With regard to the RT modality, a recent advance in preclinical RT studies that may hold particular promise for medulloblastoma translational research is the development of image-guided proton therapy for use in small animals [83,84].

Another important consideration illustrated by the studies discussed above that employed image guided CSI is the optimal enrollment threshold. Whether imaging modalities or clinical symptoms are assessed, a consensus must be reached on the most appropriate time for study initiation. It has been challenging to determine the murine parallel to intracranial tumor burden while ensuring reasonable disease progression and study timelines. Similarly, the incorporation and timing of surgical resection in the preclinical pipeline has yet to be optimized. Whereas Taylor and colleagues were able to model resection and CSI in vivo to molecularly characterize disease recurrence [74], Smith and collaborators focused on developing a protocol that would enable the incorporation of combination therapies [78]. The preclinical study design will ultimately be driven by the long-term clinical strategy and whether a given treatment is intended to be delivered up front to naïve patients or in cases of recurrence or metastasis.

Findings from our laboratories suggest that additional dose titration is needed to accomplish clinically relevant exposure while facilitating a therapeutic window for adjuvant therapy. A short-term survival advantage with subsequent recurrence of disease was achieved using 10 Gy of CSI, but the administration time (5 days) was limited when compared to the RT treatment window of approximately 6 weeks. Unpublished work by our group has evaluated the feasibility of protracted CSI dosing in several murine and PDOX models of medulloblastoma, in which the daily dose of RT is reduced to between 1 and 0.5 Gy per fraction but the cumulative CSI dosing is similar to that delivered with 2 Gy fractions. By using these lower daily doses of radiation (0.5 to 1 Gy fractions) over 2 to 4 weeks for a cumulative dose of 10 to 20 Gy, we were able to establish a therapeutic window through lifespan extension, albeit with eventual disease progression (Personal Communication, Martine F. Roussel and Christopher L. Tinkle). This protracted study design will facilitate the evaluation of concurrent systemic therapy (with DNA-damaging agents, small molecule signaling-pathway inhibitors, etc.) with CSI in the preclinical setting.

Finally, although the use of adjuvant chemotherapy has confounded the issue, it is tempting to speculate that there is a subgroup- and/or subtype-specific response to irradiation, particularly given the data from several laboratories demonstrating enhanced radiation sensitivity in medulloblastoma cells [85,86]. An early investigation of potential mechanisms governing differential RT responses in medulloblastoma subgroups suggested that *TP53* status has a significant impact on radiation response, whereas *WNT* activation may overcome this effect [87]. With the development of subgroup-specific medulloblastoma models in both GEMM and PDOX systems, preclinical testing of radiotherapy promises to inform optimal RT dosing and volume approaches that may be explored in future clinical trials, and to further delineate molecular mechanisms governing RT response and resistance in medulloblastoma.

## 5. Conclusions

MB encompasses a heterogenous group of embryonal tumors, consisting of four genetically defined subgroups with distinct molecular and clinical features and varied prognoses with current therapies. RT, targeting the entire neuraxis, represents a critically effective adjuvant therapy, yet carries the risk of significant long-term toxicities. Through the development of accurate and reproducible preclinical radiotherapy platforms modeling the clinical delivery of CSI, researchers are now poised to experimentally define optimal radiation dose-volume parameters, delineate molecular determinants of radiation response, identify radiotherapy adjuncts, and investigate normal tissue toxicity in emerging animal models systems that represent the distinct subgroups of MB. While important questions remain as to the optimal approaches of preclinical CSI, integration of this modality into translational studies offers the potential to better inform modifications to what has been the most studied treatment modality of clinical trials of MB.

## Figures and Tables

**Table 1:** Comparison of therapy and model characteristics of select commercial preclinical irradiation systems and custom approaches used to deliver craniospinal irradiation to murine models of medulloblastoma.

Characteristic	SARRP-Based Irradiation (Xstrahl Ltd., UK) [74,75]	X-RAD 225Cx Irradiation (Precision X-ray Inc., USA) [71,73]	Custom Irradiation [76]
Photon energy range	5–225 keV	5–225 keV	5–225 keV
Image guidance	Volumetric cone-beam CT	2D fluoroscopic images Volumetric cone-beam CT	Clinical set-up
Beam collimation	Motorized variable collimator	Fixed collimators	Custom CSI lead aperture
Phase of radiotherapy	Primary	Post-operative	Primary
CSI beam arrangement	Brain: arc (−90–+90°) Spine: 2 field PA	Brain: opposed laterals Spine: single or multiple PA	Planar
Boost treatment	No	No	Yes
Dose per fraction	2 Gy	Brain: 2 Gy Spine: 4.76 Gy	2 Gy
Cumulative dose	10–36 Gy	Brain: 36 Gy Spine: 28.56 Gy	20 Gy
Medulloblastoma model system	PDOX (Group 3, SHH)	GEMM (SHH)	PDOX (Group 3)
Tumor burden assessment	Bioluminescence Histologic examination	Clinical symptoms Histologic examination	Clinical symptoms Histologic examination

Abbreviations: SARRP, small animal radiation research platform; CT, computed tomography; CSI, craniospinal; PA, posterior-to-anterior beam; PDOX, patient-derived orthotopic xenograft; GEMM, genetically engineered mouse model; SHH, sonic hedgehog.

**Table 2:** Outstanding questions and considerations of preclinical modeling of medulloblastoma and its treatment.

Oustanding Questions	Considerations and Current Approaches
Clinically relevant CSI dosing regimens	Fully fractionated “human” dosing regimens vs. non-curative empiric murine regimens vs. feasible dosing regimens
Optimal enrollment threshold	Minimal tumor burden to mimic post-surgical conditions vs. moribund conditions to mimic recurrent setting; impact on RT efficacy
Tumor burden assessment	Bioluminescence imaging vs. MRI/CT vs. histopathology
Primary site tumor boost	Targeting of primary site: IGRT vs. biologic RT targeting vs. historic histopathology; integration into non-curative CSI regimens
Integration of other treatment modalities	Value of surgical resection; integration of adjuvant systemic therapy and preclinical modeling of clinically relevant systemic therapy exposure
Optimal endpoints	Overall survival vs. tumor-specific survival; tumor growth delay assays vs. tumor control dose 50% (TCD50)
Optimal MB models	GEMM vs. PDOX vs. organoids

Abbreviations: CSI, craniospinal irradiation; RT, radiation therapy; MRI, magnetic resonance imaging; CT, computed tomography; IGRT, image-guided RT; MB, medulloblastoma; GEMM, genetically engineered mouse model; PDOX, patient-derived orthotopic xenograft.
